# Hydrogen Purification through a Highly Stable Dual‐Phase Oxygen‐Permeable Membrane

**DOI:** 10.1002/anie.202010184

**Published:** 2021-02-08

**Authors:** Lujian Jia, Guanghu He, Yan Zhang, Jürgen Caro, Heqing Jiang

**Affiliations:** ^1^ Key Laboratory of Biofuels Qingdao Institute of Bioenergy and Bioprocess Technology Chinese Academy of Sciences Songling Road No.189 Qingdao 266101 China; ^2^ Institute of Physical Chemistry and Electrochemistry Leibniz University of Hannover Callinstrasse 3A 30167 Hannover Germany; ^3^ University of Chinese Academy of Sciences Beijing 100049 China

**Keywords:** hydrogen production, mixed conductor, oxygen-permeable membrane, water splitting

## Abstract

Using oxygen permeable membranes (OPMs) to upgrade low‐purity hydrogen is a promising concept for high‐purity H_2_ production. At high temperatures, water dissociates into hydrogen and oxygen. The oxygen permeates through OPM and oxidizes hydrogen in a waste stream on the other side of the membrane. Pure hydrogen can be obtained on the water‐splitting side after condensation. However, the existing Co‐ and Fe‐based OPMs are chemically instable as a result of the over‐reduction of Co and Fe ions under reducing atmospheres. Herein, a dual‐phase membrane Ce_0.9_Pr_0.1_O_2−δ_‐Pr_0.1_Sr_0.9_Mg_0.1_Ti_0.9_O_3−δ_ (CPO‐PSM‐Ti) with excellent chemical stability and mixed oxygen ionic‐electronic conductivity under reducing atmospheres was developed for H_2_ purification. An acceptable H_2_ production rate of 0.52 mL min^−1^ cm^−2^ is achieved at 940 °C. No obvious degradation during 180 h of operation indicates the robust stability of CPO‐PSM‐Ti membrane. The proven mixed conductivity and excellent stability of CPO‐PSM‐Ti give prospective advantages over existing OPMs for upgrading low‐purity hydrogen.

## Graphical abstract

By taking advantages of the high chemical stability and moderate reduction of Ti and Ce ions under reducing atmosphere, a novel Ti‐based dual‐phase oxygen‐permeable membrane was developed for H_2_ purification. The Ce_0.9_Pr_0.1_O_2−δ_/Pr_0.1_Sr_0.9_Mg_0.1_Ti_0.9_O_3−δ_ (CPO‐PSM‐Ti) membrane has excellent chemical stability and mixed oxygen ionic‐electronic conductivity under reducing atmospheres.

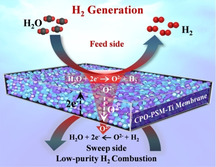

Hydrogen as a green energy shows great potentials for fulfilling the growing energy demand and addressing environmental pollution problems.^[1]^ Over the past decades, intensive efforts have been devoted to H_2_ purification technologies in order to obtain CO‐free H_2_ from industrial by‐products,^[2]^ such as coke‐oven‐gas containing H_2_, CH_4_, CO_2_ etc. In addition to Pd‐based membranes,^[3]^ H_2_ permeable dense ceramic membranes have triggered considerable research owing to their advantages of 100 % H_2_ selectivity. For example, Ce‐ or Zr‐based perovskite‐type oxide membranes ^[4]^ have been reported for H_2_ purification based on their proton conductivity at elevated temperatures. However, only a low H_2_ production rate (≤0.03 mL min^−1^ cm^−2^) was obtained due to their limited electronic conductivity. Development of dual‐phase composite membranes by adding a secondary phase as an electronic conductor has been regarded as a sound strategy to improve H_2_ production rate.^[5]^ For instance, dual‐phase membranes BaCe_0.8_Y_0.2_O_3−δ_‐Ce_0.8_Y_0.2_O_2vδ_, BaCe_0.65_Zr_0.20_Y_0.15_O_3−δ_‐Ce_0.85_Gd_0.15_O_2−δ_ have been developed as H_2_ permeable membranes by introducing doped ceria as an electronic conductor.[^2b^, ^5a^] Unfortunately, the newly formed miscellaneous phases originated from cation diffusion between two phases weaken the material stability and sorely hamper their applications.[^2b^, ^5a^] Some newly reported mixed conducting ceramic membranes such as BaCe_0.50_Fe_0.50_O_3−δ_ gave enhanced H_2_ production rate.[^5b^, ^6^]

Alternatively, a promising strategy for H_2_ purification was recently proposed using ceramic oxygen permeable membranes (OPMs), which feature mixed oxygen ionic‐electronic conductivity and possess 100 % O_2_ selectivity at elevated temperatures.^[7]^ When the two sides of the OPMs are subjected to steam and low‐purity H_2_ streams, respectively, water dissociates into H_2_ and O_2_ on the feed side, the O_2_ is removed as oxygen ions (O^2−^) through the membrane to the sweep side and there consumed by the oxidation of low‐purity H_2_ to water_._ Consequently, pure H_2_ can be obtained after steam condensation on the feed side. The H_2_ production rate based on the OPMs is normally higher than that of H_2_ permeable membrane due to their higher mixed ionic‐electronic conductivity. In our previous works,^[8]^ by coupling water dissociation with the chemical reactions such as partial oxidation of methane (POM) or oxidative dehydrogenation of ethane (ODHE) on the sweep side to consume the permeated O_2_, a high H_2_ production rate of above 1 mL min^−1^ cm^−2^ was achieved based on a BaCo_*x*_Fe_*y*_Zr_1−*x*−*y*_O_3−δ_ (BCFZ) hollow‐fiber OPM reactor. However, the Co cations in perovskite BCFZ lattice were over‐reduced under such strongly reducing atmospheres, leading to the formation of a surface eroded layer.^[8b]^ To improve the chemical stability of the membranes, many efforts have been dedicated to the development of Fe‐based OPMs, such as Sm_0.15_Ce_0.85_O_1.925_‐Sm_0.6_Sr_0.4_Al_0.3_Fe_0.7_O_3−δ_,^[7b]^ Ce_0.8_Gd_0.2_O_1.9_‐Gd_0.2_Sr_0.8_FeO_3−δ_ ^[9]^ and Ce_0.9_Pr_0.1_O_2−δ_‐Pr_0.6_Sr_0.4_FeO_3−δ_.^[10]^ Although a high H_2_ production rate and enhanced chemical stability were achieved, the obvious performance degradation and the decomposition of perovskite was inevitable due to the over‐reduction of Fe ions under the reducing atmospheres at high temperatures.^[10]^ Therefore, it is highly imperative to develop Co‐ and Fe‐free and chemically stable OPMs for H_2_ purification under harsh conditions.

In this work, a novel dual‐phase OPM with the composition of 60 mol % Ce_0.9_Pr_0.1_O_2−δ_‐40 mol % Pr_0.1_Sr_0.9_Mg_0.1_Ti_0.9_O_3−δ_ (CPO‐PSM‐Ti) was developed for H_2_ purification by employing Ti to replace Co and Fe ions. Firstly, the choice of strontium titanate is based on the fact that it shows limited ionic conductivity and good electronic conductivity due to the moderate reduction of Ti ions from +4 to +3, and also robustly maintains cubic perovskite structure under reducing atmospheres.^[11]^ Secondly, Ce_0.9_Pr_0.1_O_2−δ_ (CPO) is a well‐known oxygen‐ionic conductor and also shows limited electronic conductivity and superior chemical stability under reducing atmospheres.^[12]^ In the designed dual‐phase membrane system, the CPO and PSM‐Ti phases function as the main oxygen ionic and electronic conductors, respectively. Our experimental findings demonstrate that the CPO‐PSM‐Ti membrane possesses appropriate mixed oxygen ionic‐electronic conductivity via the moderate reduction of Ce and Ti ions under reducing atmospheres, and therefore achieve the desirable O_2_ permeation flux. Using CPO‐PSM‐Ti membrane to upgrade low‐purity H_2_ (see Figure S1), robust chemical stability was demonstrated by long‐term tests under harsh conditions.

The dual‐phase membrane CPO‐PSM‐Ti was prepared by a sol‐gel method followed by high‐temperature sintering.^[9]^ Figure 1 a shows the X‐ray diffraction (XRD) pattern of the as‐prepared membrane. It is clear that the sintered membrane consists of only CPO and PSM‐Ti phases, which coexist steadily and exhibit good chemical compatibility. Besides, the robust thermal stability of the membrane was illustrated by the in situ XRD measurements and approximate thermal expansion coefficients of two phases (Figure S2 and S3) over the temperature range of 25–900 °C. Furthermore, the detailed investigations of surface morphologies using secondary electron micrographs (SEM), backscattered electron micrographs (BSEM) and energy‐dispersive X‐ray spectroscopy (EDXS) indicate that the two phases are well‐distributed with distinct grain morphology and clear grain boundaries (Figure S4 and S5).

**Figure 1:**
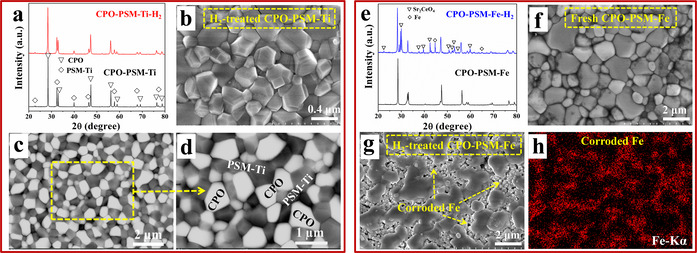
XRD results and SEM images of fresh and treated membranes. a) The XRD patterns of CPO‐PSM‐Ti membrane before (bottom) and after (top) treatment under 50 vol. % H_2_ in N_2_ at 900 °C for 10 h. b) The SEM and c),d) BSEM images of the H_2_‐treated CPO‐PSM‐Ti membrane. e) The XRD patterns of CPO‐PSM‐Fe membrane before (bottom) and after (top) treatment under 50 vol. % H_2_ in N_2_ at 900 °C for 10 h. f),g) The surface images of fresh CPO‐PSM‐Fe and H_2_‐treated CPO‐PSM‐Fe. h) The corresponding Fe element distribution.

Phase structural stability under reducing condition is a big concern for OPMs in H_2_ purification. After being treated by 50 vol. % H_2_ at 900 °C for 10 h, CPO‐PSM‐Ti membrane (annotated as CPO‐PSM‐Ti‐H_2_ in Figure 1 a) can maintain its original dual‐phase feature. The absence of chemical reduction and phase transition demonstrates its outstanding chemical stability under reducing atmospheres. Meanwhile, the robust chemical stability was also supported by the SEM analysis in Figure 1 b. It is clear that the intact crystal grains and clean grain boundaries are completely preserved after treatment under 50 vol. % H_2_. Furthermore, the BSEM images (Figure 1 c and d) present that the two phases are still evenly distributed and intimately connected after treatment by H_2_. The white grains correspond to CPO fluorite phase, while the black grains belong to PSM‐Ti perovskite phase.

For comparison, the Fe‐based dual‐phase membrane Ce_0.9_Pr_0.1_O_2−δ_‐Pr_0.1_Sr_0.9_Mg_0.1_Fe_0.9_O_3−δ_ (CPO‐PSM‐Fe) was fabricated via the similar approach. After being treated under H_2_ flow (annotated as CPO‐PSM‐Fe‐H_2_ in Figure 1 e), the original dual‐phase structure of CPO‐PSM‐Fe was seriously damaged and additional diffraction peaks attributed to Fe and Sr_2_CeO_4_ were detected. Indeed, it can be clearly seen that the intact grains of fresh CPO‐PSM‐Fe membrane (Figure 1 f) were severely corroded and the grain boundary tended to be undistinguishable after treatment by H_2_ (Figures 1 g and S6). Obviously, undesired particles precipitated from the damaged areas and the surface became porous. Furthermore, the elemental distribution of Fe (Figure 1 h) indicates that the badly corroded area belongs to PSM‐Fe phase and is resulted from the deep reduction of Fe ions. On the other hand, the CPO phase was preserved due to its high chemical stability.^[13]^ Moreover, the chemical stability of CPO‐PSM‐Ti and CPO‐PSM‐Fe membranes treated under diluted hydrogen containing some steam were also evaluated by XRD (Figure S7). Accordingly, the clear contrast in Figure 1 and Figure S7 sufficiently demonstrates the excellent chemical stability of CPO‐PSM‐Ti membrane relative to the Fe‐based membrane under reducing atmospheres.

To understand the redox chemistry of the CPO‐PSM‐Ti material, the experiments of H_2_ temperature programmed reduction (H_2_‐TPR) were performed on CPO, PSM‐Ti and CPO‐PSM‐Ti powders, respectively. As shown in Figure 2, the H_2_‐TPR of CPO powder shows two identified peaks at 439 °C and 736 °C, which can be assigned to H_2_ consumption by the surface‐capping oxygen and the lattice oxygen with the reduction of Ce^4+^ to Ce^3+^, respectively.^[14]^ The existence of cerium with mixed valence states (Ce^4+^ and Ce^3+^) implies that the oxygen‐ion conducting CPO phase also features electronic conductivity under reducing atmospheres.[^2b^, ^6a^] The PSM‐Ti sample shows only one peak at 625 °C attributed to the moderate reduction of Ti^4+^ to Ti^3+^,^[15]^ implying its electronic conductivity under reducing atmospheres. Specifically, the Ti ions can be easier reduced under reducing atmospheres owing to the lower reduction temperature relative to Ce ions (625 °C<736 °C). Based on the above study, the peak at 462 °C of CPO‐PSM‐Ti sample is ascribed to the reduction of surface‐capping oxygen in CPO phase, while a broad peak at 662 °C is attributed to the reduction of Ti^4+^ to Ti^3+^ and Ce^4+^ to Ce^3+^. The moderate reduction of Ce and Ti ions under reducing atmospheres was also illustrated by X‐ray photoelectron spectroscopy (XPS) analysis in Figure S8.[^16^, ^17^] Furthermore, the thermogravimetry (TG) analyses (Figure S9) illustrate that the CPO and PSM‐Ti phases both possess a certain amount of oxygen vacancy, which can provide the possible pathway for oxygen ion transport at elevated temperatures.[^15^, ^18^]

**Figure 2:**
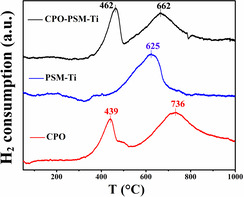
H_2_‐TPR profiles of CPO‐PSM‐Ti powder and corresponding single‐phase CPO and PSM‐Ti powders.

Prior to the H_2_ purification experiment, the O_2_ permeation flux of CPO‐PSM‐Ti membrane was evaluated under three different conditions (air//He, air//H_2_, H_2_O//H_2_). As shown in Figure 3 a, very limited O_2_ permeation fluxes (about 0.01 mL min^−1^ cm^−2^) were obtained under the air//He gradient, which may be ascribed to the lack of electronic conductivity and blocks electron transport across the membrane at oxidized atmosphere. Subsequently, when 40 vol. % H_2_ was used as sweep gas (Air//H_2_ gradient), an obvious increase in O_2_ permeation flux was observed. Firstly, the reducing H_2_ atmospheres can enhance electronic conductivity of the membrane by moderate reduction of Ti and Ce ions, as indicated by H_2_‐TPR and XPS analyses (Figures 2 and S8). Secondly, compared to the air//He condition, the higher O_2_ partial pressure gradient across the membrane was established by H_2_ combustion, which can ensure a sufficient driving force for the oxygen transport through the membrane. When H_2_O was used to replace air as the feed gas (H_2_O//H_2_ gradient), a further improvement in O_2_ permeation flux (up to 0.26 mL min^−1^ cm^−2^ at 940 °C) was observed. This result can be ascribed to the increased reducibility of the membrane materials under H_2_O//H_2_ condition. The reduction of more Ti and Ce ions results in further enhanced conductivity of the CPO‐PSM‐Ti membrane responsible for the improved O_2_ permeation fluxes. Assuming that the bulk diffusion is the rate limiting step for the oxygen permeation through the membrane, the oxygen ionic conductivity is about 7.2×10^−3^ S cm^−1^ at 940 °C calculated by Wagner equation (Figure S10 and Table S1). Meanwhile, the total conductivity of the CPO‐PSM‐Ti membrane was studied under Air or diluted H_2_ flow. As shown in Figure 3 b, the total conductivity rises with increasing temperature under the above two conditions. A higher conductivity was obtained under 15 vol. % H_2_ relative to Ar, which is mainly ascribed to the moderate reduction of Ti and Ce ions (Ti^4+^ → Ti^3+^, Ce^4+^ → Ce^3+^) under H_2_ atmospheres. The inset of Figure 3 b shows that the total conductivity increases up to 4.6×10^−2^ S cm^−1^ under a pure H_2_ atmosphere at 900 °C. Obviously, the variation of O_2_ permeation flux under different conditions (Figure 3 a) can be explained by the conductivity measurements under different atmospheres (Figure 3 b), and CPO‐PSM‐Ti membrane shows enhanced conductivity and improved O_2_ permeation flux under reducing conditions.

**Figure 3:**
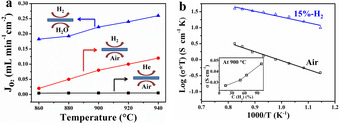
a) Oxygen permeation flux as a function of temperature under different conditions. Feed side: 40 mL min^−1^ air or 35 mL min^−1^ H_2_O diluted by 5 mL min^−1^ He. Sweep side: pure He or 40 vol. % H_2_ diluted by N_2_ (40 mL min^−1^). b) Total conductivity as a function of reciprocal temperature (600–900 °C) under air or 15 vol. % H_2_ balanced by Ar. The inset shows the total conductivity under different H_2_ concentrations at 900 °C.

The H_2_ purification experiment was carried out based on the rationally designed CPO‐PSM‐Ti membrane afterwards. As shown in Figure 4 a, H_2_ production rate on the feed side increases from 0.36 to 0.52 mL min^−1^ cm^−2^ when increasing temperature from 860 to 940 °C. This result can be ascribed as follows. (I) The rise of the temperature results in the increase of water dissociation rate and leads to larger O_2_ partial pressure gradient across the membrane. (II) High temperature can boost H_2_ combustion reaction which also enlarges the O_2_ partial pressure gradient across the membrane. (III) The increased temperature leads to enhanced conductivity of CPO‐PSM‐Ti as illustrated in Figure 3 b, and thus enhances the O_2_ removal. The H_2_ concentration and total flow rate of sweep gas also affect the H_2_ production rate, as shown in Figure S11. It should be noted that the performance of the dual‐phase membrane can be further improved by optimizing the ratio of two phases or decreasing the membrane thickness via shaping the membrane into hollow fiber or asymmetric structure.

**Figure 4:**
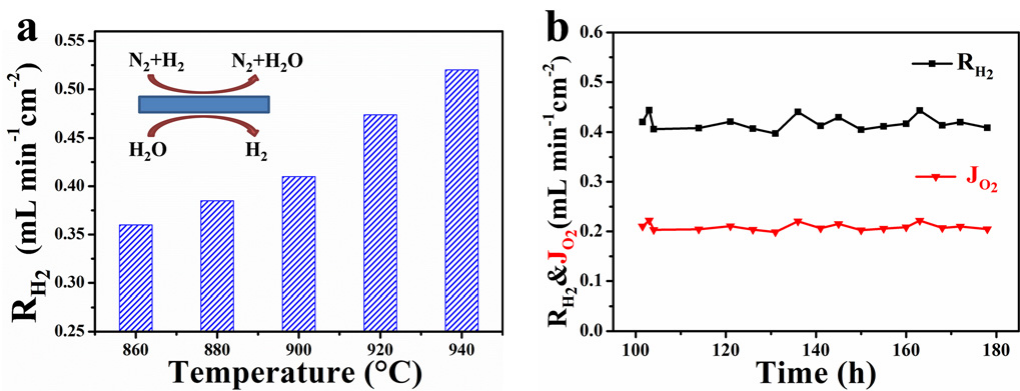
a) Effect of test temperature on the H_2_ production rate. b) Stability test of the CPO‐PSM‐Ti membrane for H_2_ purification at 900 °C. Feed side: 40 mL min^−1^ (35 mL min^−1^ H_2_O balanced by 5 mL min^−1^ He). Sweep side: 40 mL min^−1^ mixture gas (40 vol. % H_2_ diluted by N_2_).

After testing for 100 h under the above variable conditions, the membrane stability was further evaluated under the constant condition. As shown in Figure 4 b, the CPO‐PSM‐Ti membrane was stably operated without any performance degradation. The H_2_ production rate kept stable at 0.41 mL min^−1^ cm^−2^ for about 80 h before we intentionally stopped the test. No noticeable decrement in O_2_ permeation flux was found during the test, indicating that the dual‐phase oxygen permeable membrane CPO‐PSM‐Ti exhibited excellent tolerance toward reducing atmospheres.

To reveal the possible changes in crystal structure and morphology of CPO‐PSM‐Ti membrane after the purification test (180 h), XRD and SEM analyses were performed on the spent membrane. As displayed in Figure 5 a, the spent CPO‐PSM‐Ti membrane shows the similar XRD patterns on the both sides of membrane compared with the fresh membrane (Figure 1 a). The SEM images of the fractured cross‐sections of the spent membrane (Figure 5 b–d) indicated that no apparent changes occurred even after the membrane stayed under strongly reducing atmospheres at high temperature for 180 h. Moreover, no additional phases or precipitates were observed at the grain interior or boundary which is in good agreement with the XRD results (Figure 5 a). Additionally, the analyses of inductively coupled plasma optical emission spectrometry (ICP‐OES, Table S2) revealed that the spent membrane possesses similar phase composition with fresh membrane. Therefore, it can be concluded that the dual‐phase membrane CPO‐PSM‐Ti shows excellent chemical stability for upgrading low‐purity H_2_.

**Figure 5:**
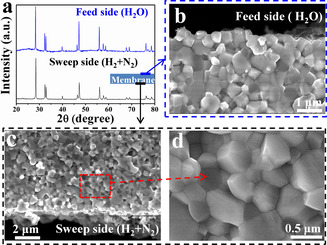
a) XRD patterns of spent CPO‐PSM‐Ti membrane after H_2_ purification test for 180 h. b) Cross‐sectional image of the membrane on feed side (H_2_O). c),d) Cross‐sectional images of the membrane on sweep side (N_2_ and H_2_) at different resolutions.

In summary, a promising dual‐phase membrane CPO‐PSM‐Ti with excellent stability was successfully developed for H_2_ purification. Comprehensive characterizations demonstrated that the CPO‐PSM‐Ti membrane exhibits mixed oxygen ionic‐electronic conductivity due to the moderate reduction of Ti and Ce ions under reducing atmospheres. Using the CPO‐PSM‐Ti membrane to purify H_2_, a H_2_ production rate of 0.52 mL min^−1^ cm^−2^ was obtained at 940 °C. Meanwhile, the excellent chemical stability was supported by the XRD and SEM analyses. The reasonably designed CPO‐PSM‐Ti membrane not only offers great potential to purify H_2_, but also presents broad prospects in other chemical and energy transformation systems.

## Conflict of interest

The authors declare no conflict of interest.

## Supporting information

As a service to our authors and readers, this journal provides supporting information supplied by the authors. Such materials are peer reviewed and may be re‐organized for online delivery, but are not copy‐edited or typeset. Technical support issues arising from supporting information (other than missing files) should be addressed to the authors.

Supplementary
