# Supplementary material for: Hydrogen Purification through a Highly Stable Dual‐Phase Oxygen‐Permeable Membrane
Source: Angew Chem Int Ed Engl. 2021 Feb 8;60(10):5204–8. doi: 10.1002/anie.202010184 (PMC7986621; doi:10.1002/anie.202010184)
Supplement: Supplementary file 1 — Supplementary [file ANIE-60-5204-s001.pdf]

Angewandte Chemie
Eine Zeitschrift der Gesellschaft Deutscher Chemiker

Supporting Information

Hydrogen Purification through a Highly Stable Dual-Phase Oxygen-Permeable Membrane

Lujian Jia, Guanghu He, Yan Zhang, Jürgen Caro, and Heqing Jiang**

anie_202010184_sm_miscellaneous_information.pdf

Table of Contents

1. Experimental Procedures 2

1.1 Materials synthesis 2

1.2 Characterizations 2

1.3 Performance test of membrane for hydrogen purification 4

2. Results and Discussion 6

Figure S1 6

Figure S2. 7

Figure S3. 8

Figure S4. 9

Figure S5. 10

Figure S6 11

Figure S7. 12

Figure S8. 13

Figure S9. 15

Figure S10 18

Figure S11 18

Table S1 16

Table S2 19

3. References 20

1. Experimental Procedures

1.1 Materials synthesis

One-step sol-gel method was used to synthesize single-phase $Ce_{0.9}Pr_{0.1}O_{2-\delta}$ (CPO), $Pr_{0.1}Sr_{0.9}Mg_{0.1}Ti_{0.9}O_{3-\delta}$ (PSM-Ti) and dual-phase 60 mol % $Ce_{0.9}Pr_{0.1}O_{2-\delta}$ - 40 mol % $Pr_{0.1}Sr_{0.9}Mg_{0.1}Ti_{0.9}O_{3-\delta}$ (CPO-PSM-Ti) and 60 mol % $Ce_{0.9}Pr_{0.1}O_{2-\delta}$ - 40 mol % $Pr_{0.1}Sr_{0.9}Mg_{0.1}Fe_{0.9}O_{3-\delta}$ (CPO-PSM-Fe) powders.[1] Different precursors were prepared by combining ethylene diamine tetra acetic acid (EDTA), citric acid and corresponding metal nitrates ($Ce(NO_3)_3$ $6H_2O$, $Pr(NO_3)_3$ $6H_2O$ $Sr(NO_3)_2$, $Fe(NO_3)_3$ $9H_2O$ and $Mg(NO_3)_2$ $6H_2O$) in an aqueous solution with appropriate stoichiometric ratios. The mole ratio of total metal cation: EDTA: citric acid was 1:1:1.5 and the pH value of mixed solution was adjusted to around 9 by adding NH_4OH solution. In order to avoid tetrabutyl titanate hydrolysis, it is necessary to dissolve it in an ethanol solution containing lactic acid and acetic acid firstly (weight ratio of tetrabutyl titanate: lactic acid: acetic acid is 1: 1: 1). Then the prepared solution was slowly added into the previous precursor solution followed by stirring for 24 h until the solution became transparent. After heat and further combustion, precursor ashes were obtained and then calcined at 950 ℃ for 10 h to remove the residual organics. The final gas-tight CPO-PSM-Ti and CPO-PSM-Fe membranes with diameter of 15 mm and thickness of 0.7 mm were prepared by compressing the above powders into disks under a pressure of 10 MPa and sintering at 1450 ℃ in ambient air for 10 h. The CPO-PSM-Ti coating paste was printed on the membrane surface with a brush. The coated membrane was fired at 1200 ℃ for 1 h to improve the adhesion between coating layer and membrane.

1.2 Characterizations

The crystal structures of different powders and membranes were studied using X-ray diffraction (XRD, D8 Advance, Bruker-AXS, with Cu Kα radiation) at 2θ through the range of 20°-80° with intervals of 0.02°. In situ XRD for CPO-PSM-Ti membrane was performed using a high temperature cell (Buehler HDK 2.4 with REP 2000), with steps of 100 ℃ at 12 ℃ min^{-1} heating rate in the temperature range 25-900 ℃. To observe the surface morphology, grain boundary and cross section of

fresh and spent membranes were investigated by scanning electron microscopy (SEM, Hitachi S-4800). The backscattered electron micrographs and energy-dispersive X-ray (EDXS) analyses were characterized by a Thermo Scientific Prisma E scanning electron microscope (SEM). To compare the chemical stability, the CPO-PSM-Ti and CPO-PSM-Fe membranes were treated under 50 vol. % H_2 in dry or wet N_2 (total flow rate was 60 mL min^{-1}) at 900 ℃ for 10 h.

The XPS of CPO-PSM-Ti powder was tested by a X-ray photoelectron spectroscopy equipped with Ar ion sputtering gun (~ 3 keV) with a monochromatic Al Kα X-ray source of 1486.6 eV under a background vacuum of 2.0×10^{-7} Pa at room temperature. Before XPS test, the CPO-PSM-Ti powder was treated by 50 vol. % H_2 mixture gas (diluted by 30 mL min^{-1} Ar) at 900 ℃ for 10 h. The charge correction was done using the carbon 1s peak (binding energy = 284.6 eV) as the reference. The XPS data was fitted by Avantage software. Hydrogen temperature programmed reduction (H_2-TPR) was performed in chemical adsorption instrument (AutoChem 2920 II) with a quartz microreactor, and about 100 mg sample was used in each measurement. The samples were firstly treated under a He flow at 200 ℃ for 1 h to remove the adsorbed moisture and cooled down to 50 ℃. The hydrogen was then switched into the system, following the samples were heated up to 1000 ℃ from 50 ℃ at a rate of 10 ℃ min^{-1}. The change of hydrogen concentration was monitored by the thermal conductivity detector (TCD) of the instrument. The oxygen release property of CPO and PSM-Ti was evaluated by a flow microbalance (STA449F5 Jupiter). For the experimental measurement, about 60 mg powder was placed in an alumina holder. The sample was pretreated in a flow of Ar at 110 ℃ to remove the moisture until a stable weight value was achieved. Then temperature increased to a desired value (e.g., 900 ℃) at a heating rate of 10 ℃ min^{-1} in Ar (20 mL min^{-1}) atmosphere. The sample weight was monitored under flowing Ar with the thermogravimetric (TG) analysis kinetic curves. The actual cations concentrations of fresh and spent membranes were detected by Agilent ICP-OES 730.

The electrical conductivity measurement for the sintered membrane was carried out using Interface 5000 E potentiostat / galvanostat (GAMRY Instruments) at 600-940 ℃. Both sides of membrane were grounded and polished to have the desired thickness and flat surfaces. The gold paste was firstly

brushed on the both surfaces of the membrane as the electrodes and dried at 200 ℃-10 min, followed by calcination at 800 ℃ for 2 h. The data was acquired using a two-electrode cell configuration ensuring equilibrium conditions at each point. Electrical conductivities were tested under pure argon and hydrogen with different concentrations, respectively. The system was equilibrated under each condition until reaching the steady state, and then the first data point was taken.

1.3 Performance test of membrane for hydrogen purification

The oxygen permeation tests and hydrogen purification experiments were carried out using a self-made high temperature device as described in our previous work.[1] The membrane was fixed on an alumina tube and sealed using a glass ring (Schott 8252) at 1053 ℃ for 1 h. The operation temperature was controlled by a microprocessor temperature controller equipped with a thermocouple. The effective area of the membrane was about 0.6 cm^2. All gas flows (He, N_2, H_2) were controlled by gas mass flow controllers. The flow rate of steam was controlled by a liquid mass flow controller, and water had been fully evaporated at 180 ℃ before feeding into the reactor. All gas mass flow controllers were accurately calibrated with a soap flow meter and liquid mass flow controller was calibrated by measured the water weight among given time. The compositions of the outlet gases after condensation were analyzed by a gas chromatograph (GC, Agilent 7890B equipped with Porapak Q and 13X columns) using TCD. When synthetic air and He were used as the feed gas and sweep gas, respectively, the oxygen permeation flux was calculated according to the equation (1). Only when the leakage of oxygen was less than 5 % under air/He atmosphere, additional permeation measurements under air/H_2 and H_2O/H_2 atmospheres were then carried out.

$$J_{O_2} = \left(C_{O_2} - \frac{C_{N_2}}{4.02}\right) \times \frac{F_{total,out}}{A} \quad (1)$$

Where, C_{O2} and C_{N2} are the oxygen and nitrogen concentrations on the sweep side. F is the total flow rate of the outlet and A is the effective membrane area. 4.02 is the Knudsen diffusion factor of nitrogen and oxygen leakage through possible pores or cracks, and Knudsen diffusion factor was calculated by following equation (2).

$$J_{N_2}^{Leak}:J_{O_2}^{Leak} = \sqrt{\frac{M_{O2}}{M_{N2}}} \times \frac{0.79}{0.21} = 4.02 \quad (2)$$

When the diluted H_2 was used as the sweep gas, the oxygen permeation flux was calculated according to the equation (3).

$$J_{O_2} = \frac{F_{H_2,inlet} - F_{H_2,out}}{2A} \quad (3)$$

When hydrogen purification experiments were conducted, feed side was mixed gas (35 mL min^{-1} steam balanced by 5 mL min^{-1} He) and sweep gas was mixed H_2 and N_2. The different H_2 concentrations on sweep side were adjusted by controlled the flow rate of H_2 and N_2. The H_2 production rate was tested under different temperatures, H_2 concentrations and total flow rates of sweep gas. The H_2 production rate and oxygen permeation flux were calculated as the following equations (4), (5).

$$R(H_2\,sep) = \frac{(C_{H2} - C_{leak}) \times F_{total,out}}{A} \quad (4)$$

$$J_{O_2} = \frac{1}{2} R(H_2\,sep) \quad (5)$$

Where, C_{H2} is the hydrogen concentrations on the feed side detected by GC. C_{leak} means the leaked hydrogen through possible pores or cracks that was calculated according to equation (6).

$$C_{leak} = \sqrt{\frac{M_{N2}}{M_{H2}}} \times \frac{F_{H2}}{F_{N2}} \times C_{N_2} = 3.74 \times \frac{F_{H2}}{F_{N2}} \times C_{N_2} \quad (6)$$

M stands for the relative molecular mass, C stands for the gas concentration on the feed side and F stands for the flow rate of the sweep gas. N_2 concentration in the feed side was detected by GC. The H_2 leakage was subtracted for calculating the H_2 production rate.

2. Results and Discussion

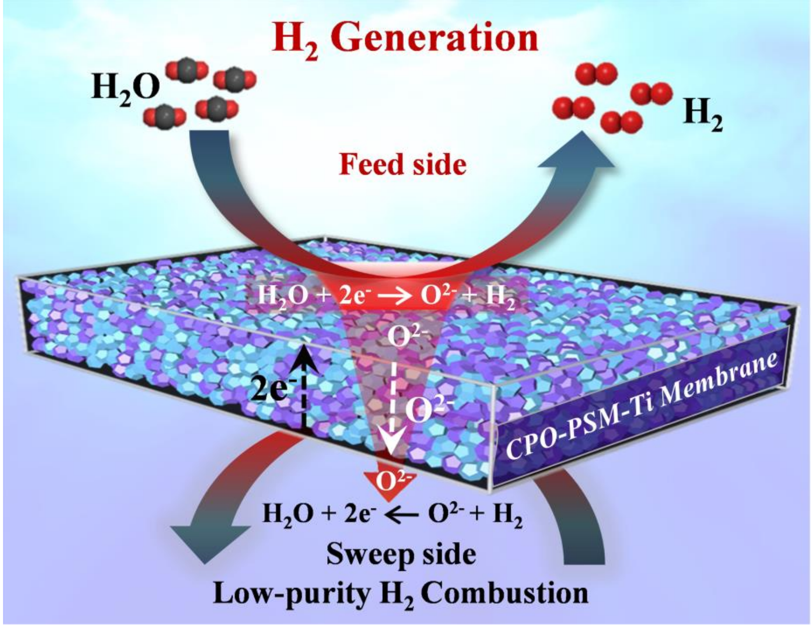

Figure S1. The schematic illustration of hydrogen purification using CPO-PSM-Ti oxygen permeable membrane.

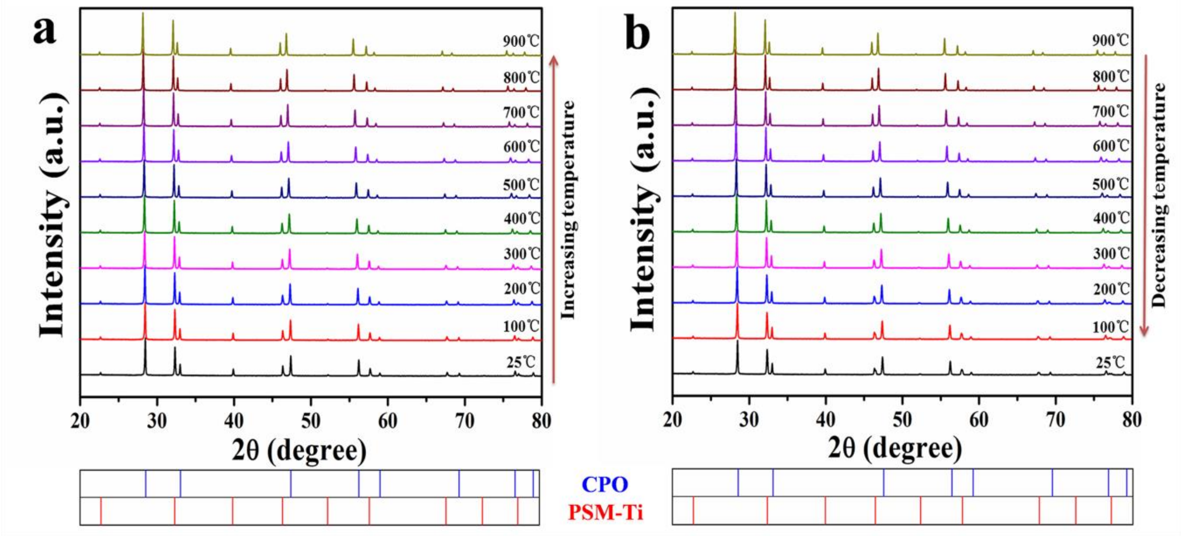

Figure S2. In situ XRD patterns of the CPO-PSM-Ti membrane sintered at 1450 °C in the (a) heating and (b) cooling mode. Conditions: heating rate was 12 °C min^{-1}; equilibration time 40 min for recording the XRD data at each temperature.

The thermal stability of CPO-PSM-Ti was investigated by in situ XRD measurements over the temperature range of 25-900 °C with steps of 100 °C under air atmosphere, as displayed in Figure S2. In the heating and cooling mode, the XRD patterns of CPO-PSM-Ti show dual phase with high crystalline degree. The main diffraction peaks assigned to the fluorite and perovskite phases were maintained up to 900 °C. With increasing temperature, the diffraction peaks shifted progressively to lower 2θ angle, a sign of lattice expansion. In the same manner, the diffraction peaks shifted progressively to higher 2θ angle and implied the lattice shrinkage with the temperature decreasing.

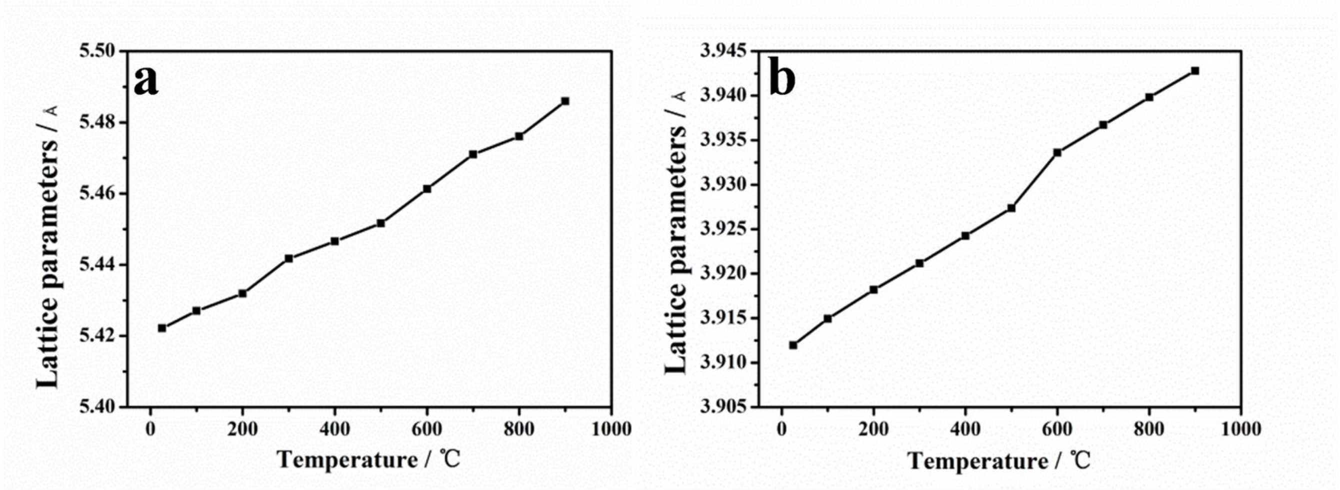

Figure S3. Lattice parameters evolutions of (a) CPO and (b) PSM-Ti as a function of the temperature. The data were calculated from the in situ XRD data (Figure S2)

Figure S3 shows the variations of the lattice parameters with increasing temperature calculated from the in situ XRD results. The thermal expansion coefficients (TEC) of CPO and PSM-Ti phases are 3.41×10^{-5} K^{-1} and 2.73×10^{-5} K^{-1}, respectively. The cracking caused by thermal expansion mismatch of two phases can be effectively inhibited due to the approximate thermal expansion coefficients for CPO and PSM-Ti phases.

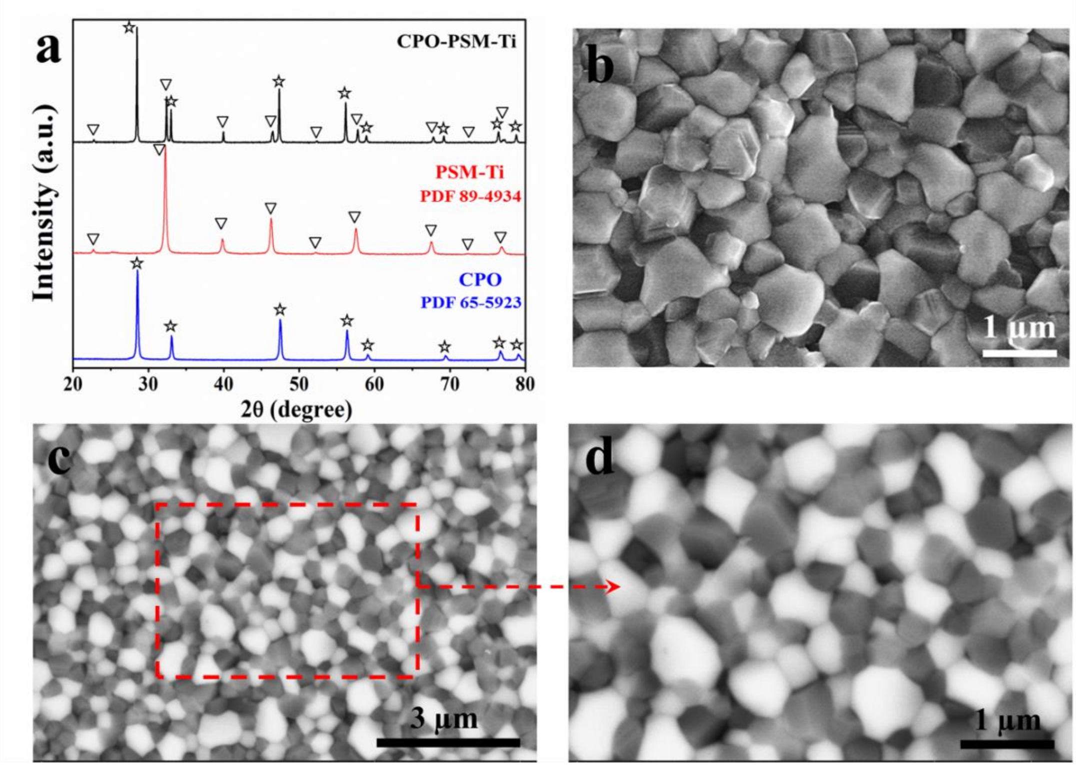

Figure S4. (a) XRD patterns of CPO, PSM-Ti and CPO-PSM-Ti powders calcined at 950 °C for 10 h; (b) SEM image of fresh CPO-PSM-Ti membrane; (c-d) BSEM images of fresh CPO-PSM-Ti membrane at different magnifications.

Shown in Figure S4a is the crystal structure of as-prepared CPO, PSM-Ti and CPO-PSM-Ti powders. CPO diffractogram is perfectly indexed to the reflections of a cubic crystal system in the *Fm-3m* space group (JPCD 65-5923) while PSM-Ti diffractogram matches well to a standard cubic crystal system, *Pm-3m* space group (JPCD 89-4934). All the diffraction peaks of CPO-PSM-Ti can be assigned to either CPO or PSM-Ti phases, indicating that the two phases co-exist in the CPO-PSM-Ti composite with good chemical compatibility. The morphology of the sintered CPO-PSM-Ti membrane (Figure S4b) depicts the compact grains and clear grain boundary, indicating that the sintered membrane is dense without evident cracking and delamination. The back scattered SEM (BSEM) images of the membrane (Figure S4c and d) show that the two phases are well-distributed and form a 3-dimensional percolation network.

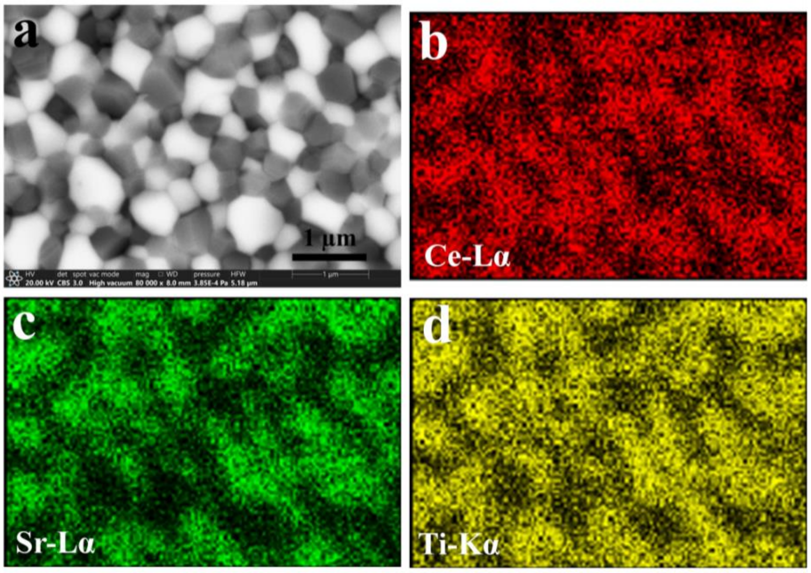

Figure S5. (a) BSEM and (b-d) EDXS images of the dual-phase CPO-PSM-Ti membrane after being sintered at 1450 °C for 10 h in air.

Figure S5 shows the detailed information of crystal structure by energy dispersive X-ray spectroscopy (EDXS). The EDXS elemental distributions of Ce in the CPO grains (Figure S5b) and of Sr and Ti in the PSM-Ti grains (Figure S5c and d) indicate the phase separation in the membrane and show that no intermixing of Ce and Sr between the two phases exists, while the white grains are CPO and the black grains are PSM-Ti.

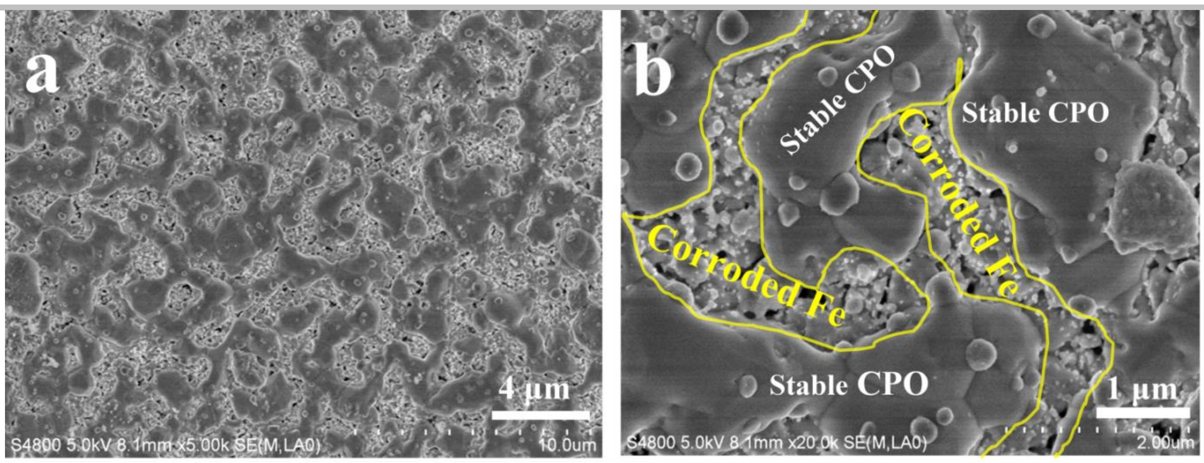

Figure S6. SEM images of CPO-PSM-Fe after treatment under dry 50 vol. % H_2 at 900 °C for 10 h.

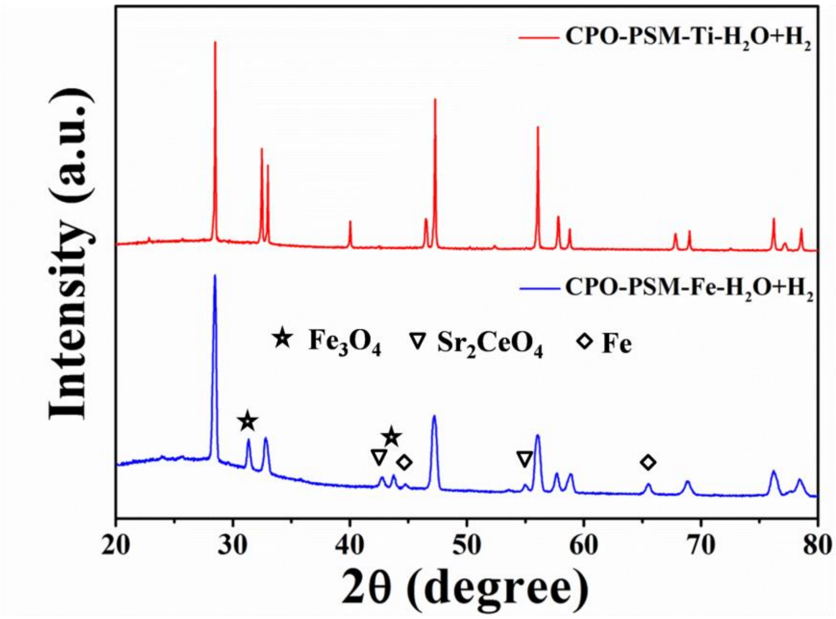

Figure S7. XRD patterns of CPO-PSM-Ti and CPO-PSM-Fe membranes treated under wet 50 vol. % H_2 in N_2 at 900 ℃ for 10 h.

The CPO-PSM-Ti and CPO-PSM-Fe membranes were treated in diluted hydrogen flow containing some steam. As shown in Figure S7, the treated CPO-PSM-Ti consists of only CPO and PSM-Ti phases, while the CPO-PSM-Fe membrane has been obviously destroyed after the treatment.

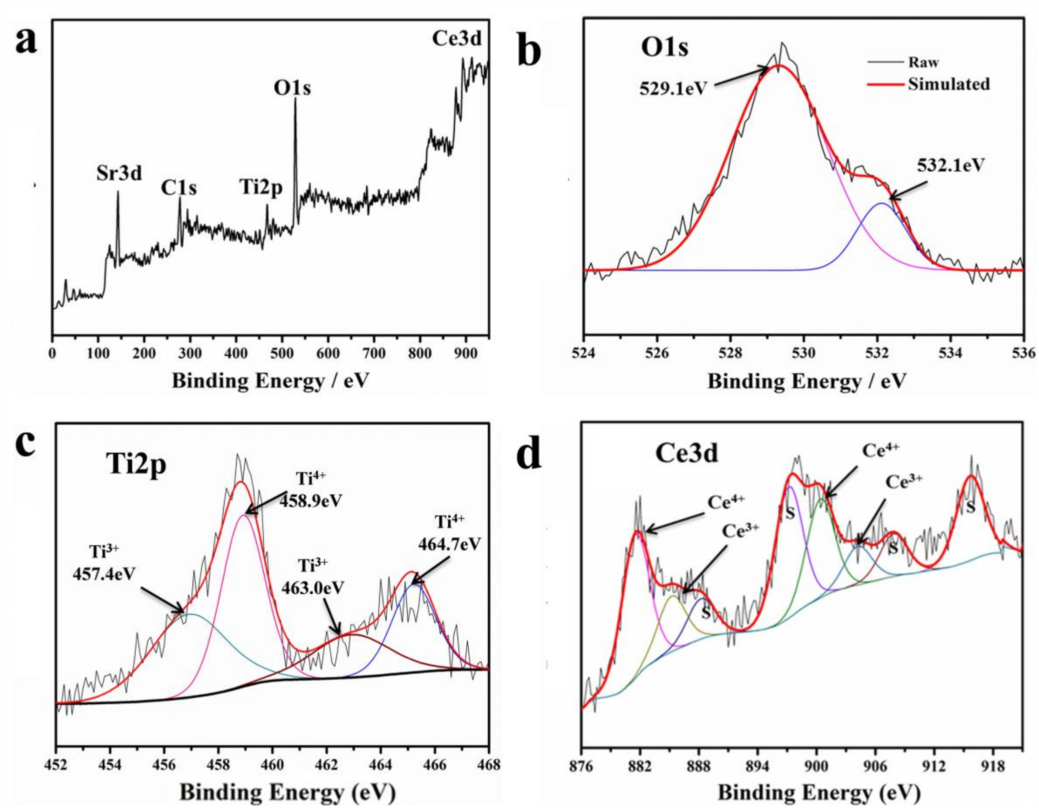

Figure S8. (a) The XPS survey spectrum of the CPO-PSM-Ti powder after reduction under 50 vol. % H_2 at 900 °C for 10 h; (b-d) XPS spectra of O 1s, Ti 2p and Ce 3d regions.

The characteristic peaks of Sr, C, Ti, O and Ce can be obviously observed in the survey spectrum (Figure S8a). The XPS of O 1s spectrum is shown in Figure S8b from which the deconvolution peaks centred at 529.1 eV and 532.1 eV correspond to the definite oxygen species.[2] The peak at 529.1 eV is ascribed to the lattice oxygen and the one at 532.1 eV is attributed to surface adsorbed oxygen. Figure S8c displays the high-resolution XPS spectrum of Ti 2p in the CPO-PSM-Ti sample. The fitting results of the experimental data show two peaks at the binding energy of 458.8 eV and 465.1 eV, which point to the Ti 2p3/2 and Ti 2p1/2, respectively.[3] Especially, the Ti 2p3/2 peak is well fitted by the two peaks of Ti^{4+} (458.9 eV) and Ti^{3+} (457.4 eV) and the Ti 2p1/2 peak is well fitted by the two peaks of Ti^{4+} (464.7 eV) and Ti^{3+} (463.0 eV), clearly demonstrating that the Ti ions became reduced by H_2. It is worthy to mention that there are no Ti^{2+}, Ti^{+} and metallic Ti species at the surface of sample, indicating that Ti ions are only modestly reduced from Ti^{4+} to Ti^{3+} without over-reduction, and thus cubic-phase

crystalline structure can be maintained. Figure S8d depicts the XPS spectra of Ce 3d, whose fitting results present four peaks at 881.6 eV, 885.4 eV, 900.5 eV and 904.3 eV, corresponding to Ce^{4+}3d 5/2, Ce^{3+}3d 5/2, Ce^{4+}3d 3/2 and Ce^{3+}3d 3/2, respectively.[4]

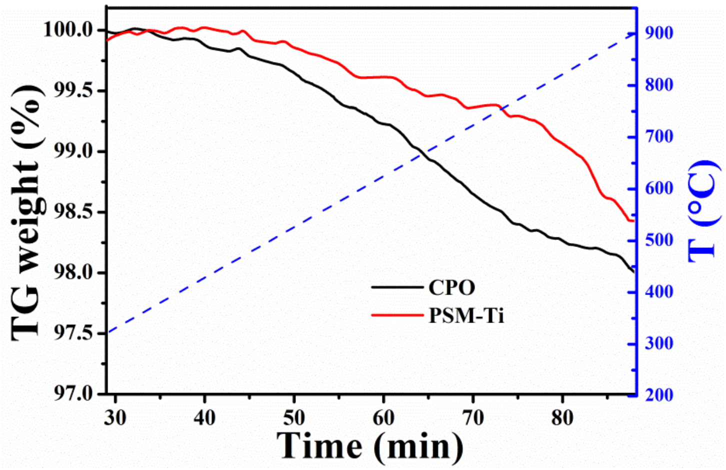

Figure S9. Thermogravimetry analyses of CPO and PSM-Ti powders.

As shown in Figure S9, about 2.0 wt. % weight loss was observed for CPO as temperature rise from 300 °C to 900 °C, while the weight loss for PSM-Ti sample was only about 1.5 wt. %. The higher weight loss implied that more available lattice oxygen was released from CPO phase, indicating that the CPO has higher oxygen vacancy concentration under low oxygen partial pressure.[5] Meanwhile, the PSM-Ti also shows oxygen release ability at elevated temperatures, which may be ascribed to the low-valence Mg^{2+} doping [6] or the change in Ti valence states according to Equation: Ti^{4+}-(O^{2-})-Ti^{4+}=Ti^{3+}-□-Ti^{3+}+1/2O_2 ("□" represents oxygen vacancy) at low O_2 partial pressure.

Table S1. The calculated equilibrium P_{O2} on the two sides of membrane and the oxygen ionic conductivity (σ_{ion}).

Temp. (°C)	P_{O2} at feed side (atm)	P_{O2} at sweep side (atm)	σ_{ion} (S cm^{-1})
860	1.49E-13	2.12E-21	0.0050
880	3.34E-13	5.9E-21	0.0052
900	6.04E-13	1.92E-20	0.0062
920	1.22E-12	5.27E-20	0.0066
940	2.37E-12	1.42E-19	0.0072

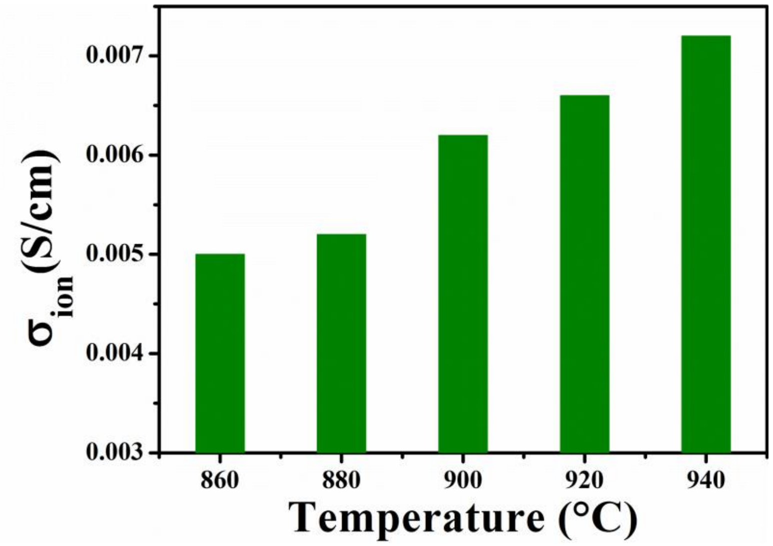

Figure S10. The oxygen ionic conductivity of CPO-PSM-Ti membrane as a function of temperature calculated based on the oxygen permeation fluxes in Figure 3a.

Considering the membrane surface has been activated by porous layer and the thickness of membrane is large enough, we assume that the bulk diffusion is the rate limiting step for oxygen permeation through the membrane. Therefore, according to Wagner theory, the oxygen permeation flux (J_{O2}) during the permeation process can be written as the following Equation (1).

$$J_{O2} = \frac{RT}{16F^2L}\frac{\sigma_{\text{ion}}\sigma_{el}}{\sigma_t} ln(\frac{P'_{O2}}{P''_{O2}}) \tag{1}$$

where R is the gas constant, L is the membrane thickness, F is the Faraday's constant, σ_t is the total conductivity, σ_{el} and σ_{ion} are the electronic conductivity and oxygen-ionic conductivity, respectively. P'_{O2} and P''_{O2} are the O_2 partial pressure of feed and sweep sides, respectively. The oxygen ionic

conductivity (σ_{ion}) of a mixed conductor can be calculated from the oxygen permeation flux by integrating Equation (1), thus modifying the final form to Equation (2).[7-8]

$$\sigma_{\mathrm{ion}} = J_{\mathrm{O2}} \frac{16F^2L}{RT} / ln(\frac{P'_{O2}}{P''_{O2}}) \qquad (2)$$

The oxygen permeation fluxes of CPO-PSM-Ti membrane under a condition of $H_2O//H_2$ (Figure 3) were used to calculate the σ_{ion}. All the equilibrium compositions on the two sides are calculated using Gibbs free energy minimization simulations using HSC Chemistry 5.0. Table S1 shows the thermodynamic equilibrium P_{O2} on two sides of the membrane and the oxygen ionic conductivity (σ_{ion}) at different temperatures (860-940 °C) at 1 atm. As shown in Figure S10, the oxygen ionic conductivity of membrane calculated by Equation (2) increases from 0.005 S cm^{-1} at 860 °C to 0.0072 S cm^{-1} at 940 °C.

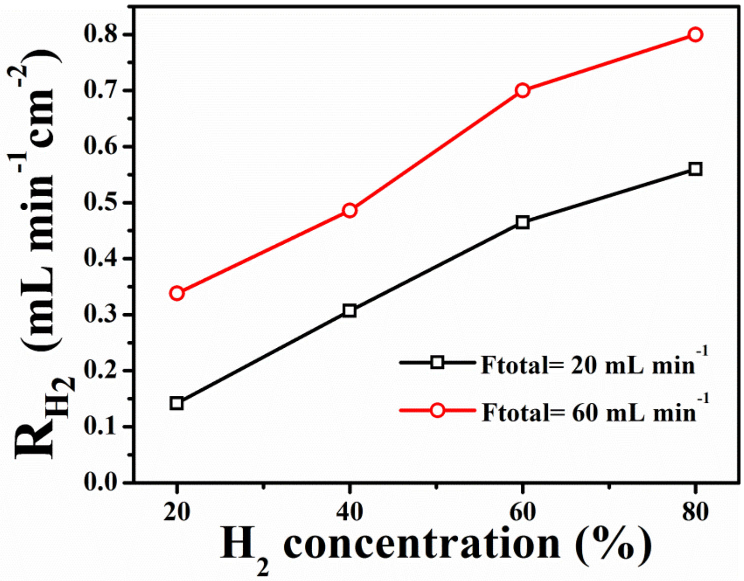

Figure S11. Effects of H_2 concentration on the H_2 production rate at 900 °C. Feed side: 35 mL min^{-1} H_2O balanced by 5 mL min^{-1} He. Sweep side: low-purity H_2 in different concentrations with total flow rates of 20 and 60 mL min^{-1}.

As shown in Figure S11, when low-purity hydrogen (H_2 and N_2) with different flow rates (20 or 60 mL min^{-1}) was used to consume the permeated O_2 under the condition of 40 mL min^{-1} (H_2O+He) as feed gas, the H_2 production rate gradually increased with the increase of H_2 concentration on the sweep side. An increase of H_2 production rate from 0.33 to 0.80 mL min^{-1}cm^{-2} was obtained with the increase of H_2 concentration from 20 vol. % to 80 vol. % using 60 mL min^{-1} H_2 and N_2 as sweep gas.

Table S2. The weight percentages of Ce, Pr, Sr, Ti and Mg cations in fresh and spent membranes.

Cations	Ce (wt%)	Pr (wt%)	Sr (wt%)	Ti (wt%)	Mg (wt%)
Nominal value	42.5	7.9	17.7	9.7	0.54
Fresh membrane	42.5	8.5	20.5	9.4	0.43
Spent membrane	40.6	8.3	19.4	9.2	0.43

As shown in Table S2, the nominal weight percentages (wt%) of Ce, Pr, Sr, Ti and Mg cations are 42.5%, 7.9%, 17.7%, 9.7% and 0.54%, respectively. In order to quantify the phase composition of fresh and spent membranes, inductively coupled plasma optical emission spectrometry (ICP-OES) analysis was conducted to monitor the chemical composition of the membrane. Table S2 shows the actual elemental compositions of fresh and spent membranes.

3. References

[1] W. Liang, Z. Cao, G. He, J. Caro, H. Jiang, *ACS Sustainable Chem. Eng.* **2017**, *5*, 8657-8662.

[2] D. Chen, D. He, J. Lu, L. Zhong, F. Liu, J. Liu, J. Yu, G. Wan, S. He, Y. Luo, *Appl. Catal. B* **2017**, *218*, 249-259.

[3] G. Li, J. Li, G. Li, G. Jiang, *J. Mater. Chem. A* **2015**, *3*, 22073-22080.

[4] C. Paun, O. V. Safonova, J. Szlachetko, F. Krumeich, J. A. Bokhoven, *J. Phys. Chem. C.* **2012**, *116*, 7312-7317.

[5] Y Zhang, H Jiang, *Chem. Commun.* **2018**, *54*, 10343-10346.

[6] G. He, Z. Cao, W. Liang, Y. Zhang, X. Liu, J. Caro, H. Jiang, *Ind. Eng. Chem. Res.* **2016**, *55*, 10386-10393.

[7] C. Zhou, J. Sunarso, Y. Song, J. Dai, J. Zhang, B. Gu, W. Zhou, Z. Shao, *J. Mater. Chem. A* **2019**, *7*, 13265-13274.

[8] S. K. Kim, M. J. Shin, J. Rufner, K. Benthem, J. H. Yu, S. Kim, *J. Membr. Sci.* **2014**, *462*, 153-159
